# Analysis of a Cantilevered Piezoelectric Energy Harvester in Different Orientations for Rotational Motion

**DOI:** 10.3390/s20041206

**Published:** 2020-02-22

**Authors:** Wei-Jiun Su, Jia-Han Lin, Wei-Chang Li

**Affiliations:** 1 Department of Mechanical Engineering, National Taiwan University, No. 1, Sec. 4, Roosevelt Rd., Taipei 10617, Taiwan; 2 Institute of Applied Mechanics, National Taiwan University, No. 1, Sec. 4, Roosevelt Rd., Taipei 10617, Taiwan

**Keywords:** piezoelectric, energy harvesting, rotational motion, passive tuning

## Abstract

This paper investigates a piezoelectric energy harvester that consists of a piezoelectric cantilever and a tip mass for horizontal rotational motion. Rotational motion results in centrifugal force, which causes the axial load on the beam and alters the resonant frequency of the system. The piezoelectric energy harvester is installed on a rotational hub in three orientations—inward, outward, and tilted configurations—to examine their influence on the performance of the harvester. The theoretical model of the piezoelectric energy harvester is developed to explain the dynamics of the system and experiments are conducted to validate the model. Theoretical and experimental studies are presented with various tilt angles and distances between the harvester and the rotating center. The results show that the installation distance and the tilt angle can be used to adjust the resonant frequency of the system to match the excitation frequency.

## 1. Introduction

Piezoelectric energy harvesting has attracted great attention in the past decade because of the demand for self-powered electronics, such as sensors [1,2,3,4], pacemakers [5,6], and mobile devices [7]. Several review papers have examined the development and evaluated the feasibility of using piezoelectric energy harvesting as an energy source for electronics [8,9]. A conventional piezoelectric energy harvester (PEH) has a cantilever structure with one or two piezoelectric patches attached for power generation. Many studies have been conducted to study the cantilevered PEH under base excitations. The mathematical model has been deeply discussed in the literature [10,11,12,13,14]. Although a cantilevered PEH has the advantage of simplicity, its resonant frequency needs to be properly tuned to match the excitation frequency in order to achieve high power output. In order to improve performance, different techniques have been adopted to achieve frequency tuning for piezoelectric energy harvesting from rectilinear excitations. Hu et al. [15] and Eichhorn et al. [16] utilized a screw to adjust the axial preload of a PEH to tune its resonant frequency. Magnetic force [17,18,19] has also been used to change the equivalent stiffness of PEHs. Alternatively to mechanical tuning, Moral et al. [20] and Brenes et al. [21] utilized the synchronized electrical charge extraction technique to perform frequency tuning for a strongly coupled piezoelectric energy harvester.

Besides scavenging kinetic energy from base excitations, PEHs can also be used to convert kinetic energy from rotational motion into electrical energy. Rotational PEHs can be categorized into two types. The first type contains a stator and a rotating hub, and these two components are coupled by magnetic force [22,23,24,25,26] or contact force [27]. When the rotating hub runs, reciprocating force will be generated to excite the PEH. Several studies [22,23,24] achieved energy harvesting from human motion by attaching a magnet on a freely rotating eccentric mass and relied on the magnetic force to pluck a piezoelectric cantilever with a tip magnet. Fu and Yeatman [25] discussed the influence of the arrangement of the rotating magnets and the beam on the performance of the PEH. Shu et al. [26] analyzed a rotary magnetic plucking PEH with a rectifier circuit. The other type of rotational energy harvesting is to mount a PEH on a horizontal rotating hub. As the hub rotates, the direction of the gravitational force varies with respect to the hub to excite the PEHs [28,29,30,31,32,33,34,35,36,37,38,39,40,41,42]. Although the excitation force caused by the gravity under rotational motion is similar to that caused by rectilinear excitations, the centrifugal force may affect the mechanical characteristics of the rotational PEHs. Gu et al. [28] and Hsu et al. [29] proposed an outwardly installed cantilevered PEH that used a tip mass at the free end for rotational energy harvesting. The resonant frequency of the PEH was passively tuned by the centrifugal force that changed with the rotational speed. Rui et al. [30] determined a design parameter for the outwardly installed rotational cantilevered PEH to optimize the self-tuning effect and achieve broadband harvesting. Guan and Liao [31] installed the PEH in the inward orientation and made the tip mass close to the rotating center to achieve a small centrifugal force. Instead of directly exciting the piezoelectric beam, Gu and Livermore [32] proposed a PEH composed of a driving beam with a tip mass and a driven piezoelectric beam. The driving beam was excited by the rotational motion and its tip hit the piezoelectric beam to generate energy. Wang et al. [33] proposed a self-tuning PEH for rotational motion. The resonant frequency could be adjusted by changing the effective length of the beam due to centrifugal force under rotational motion. Hsieh et al. [34] developed the theoretical model of a rotational PEH in outward, inward, and transverse configurations to examine their impact on the resonant frequency of the PEH. Instead of frequency tuning, Sadeqi et al. [35] proposed a broadband PEH by attaching a spring-mass system to a piezoelectric beam. By properly tuning the resonant frequency of the spring-mass system, the first two resonant frequencies of the PEH became close enough to form a broad bandwidth. Febbo et al. [36] and Ramírez et al. [37] utilized a multi-degree-of-freedom PEH composed of a multibeam structure to achieve low-frequency rotational energy harvesting. Bistable PEHs consisting of a piezoelectric cantilever beam and a pair of magnets were presented for rotational motion and showed the capability of broadband harvesting [38,39,40,41]. Zou et al. [42] developed a rotational PEH that consisted of two beams coupled by magnetic force to improve the bandwidth.

In the literature, a cantilevered rotational PEH in the inward and outward configurations at different installation distances has been investigated. However, there are no studies of the relationship between the installation angle and the dynamic characteristics of the rotational PEH. In this paper, we analyze the influence of the installation orientation of the rotational PEH on its performance. The theoretical model is derived and experimentally verified. This paper is organized as follows. The configurations of the PEH and its model are presented in Section 2, as well as the experimental set-up. The theoretical and experimental results are depicted in Section 3. Finally, conclusions are drawn in Section 4.

## 2. Materials and Methods

**Figure 1:**
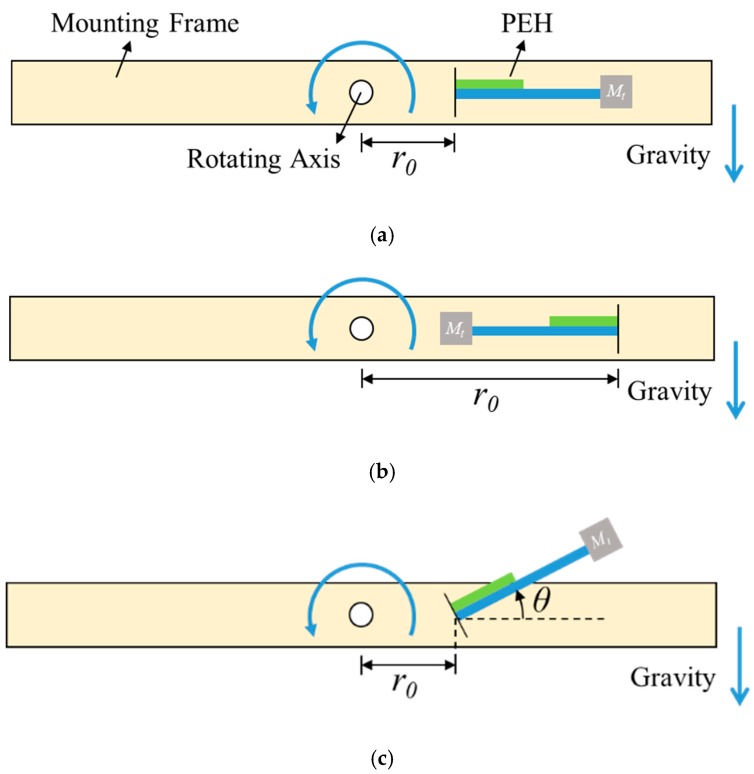
The piezoelectric energy harvester (PEH) under rotational motion in different orientations: (**a**) the outward configuration, (**b**) the inward configuration, and (**c**) the tilted configuration.

Figure 1 shows the PEH in three different orientations: outward, inward, and tilted configurations. The orientation of the PEH influences the direction of the centrifugal force acting on the beam. Note that the outward and inward configurations are the tilted configuration when *θ* is 0° and 180°, respectively. Therefore, the derivation of the model focused on the tilted configuration. The distance between the rotating center and the fixed end of the PEH is represented as *r*_0_.

### 2.1. Modeling of the Proposed PEH

**Figure 2:**
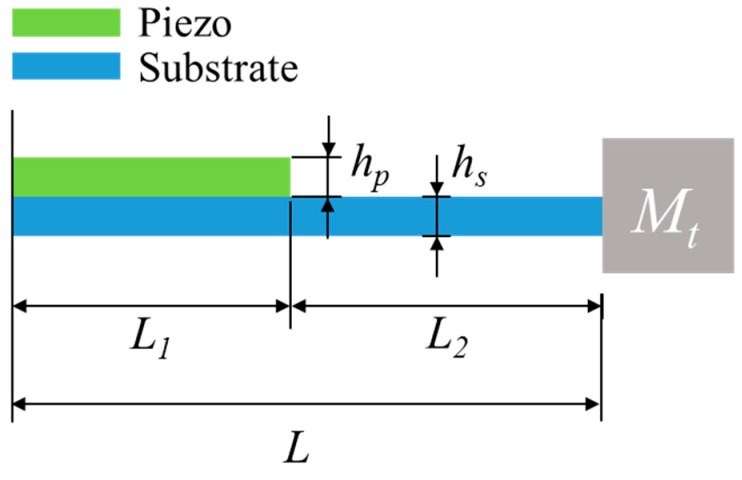
Schematic of the PEH.

**Figure 3:**
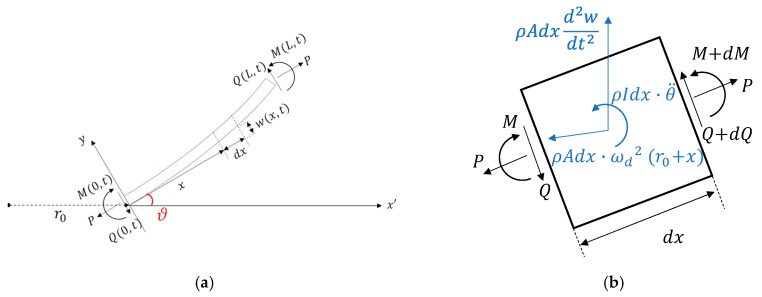
Force diagrams of (**a**) the whole beam (**b**) an infinitesimal element.

The schematic of the PEH is shown in Figure 2. The PEH was a cantilevered beam with a partially covered piezoelectric layer. A tip mass was attached to the free end of the beam. The beam is was considered as a two-segment structure. The force and moment acting on the beam in the tilted configuration and an infinitesimal element of the beam is illustrated in Figure 3. The model is based on Euler–Bernoulli beam theory. Considering the inertial forces and the electromechanical coupling term, the equation of motion can be written as:

(1) $$EI_{1}\frac{\partial^{4}w_{1}}{\partial {x_{1}}^{4}}-P\frac{\partial^{2}w_{1}}{\partial {x_{1}}^{2}}+m_{1}\frac{\partial^{2}w_{1}}{\partial t^{2}}-m_{1}\omega_{d}^{2}w_{1}+\varphi_{v}v(t)[\frac{d\delta (x_{1})}{dx_{1}}-\frac{d\delta (x_{1}-L_{1})}{dx_{1}}]=0$$

(2) $$EI_{2}\frac{\partial^{4}w_{2}}{\partial {x_{2}}^{4}}-P\frac{\partial^{2}w_{2}}{\partial {x_{2}}^{2}}+m_{2}\frac{\partial^{2}w_{2}}{\partial t^{2}}-m_{2}\omega_{d}^{2}w_{2}+m_{2}\omega_{d}^{2}r_{0}\sin\theta =0$$

where the subscript indicates the segment number. The first segment was covered with a piezoelectric layer, while the second segment was not; *EI* is the bending stiffness; *w* is the transverse displacement of the beam; *m* is the mass per unit length; *ω_d_* is the rotational speed of the motor; *L*_1_ is the length of the first segment; *r*_0_ is the distance between the fixed end of the beam and the rotating axis; *θ* is the tilt angle of the beam; *P* is the axial load due to the centrifugal force caused by the tip mass and can be expressed as:

(3) $$P=M_{t}\omega_{d}^{2}(r_{0}\cos\theta +L)$$

where *M_t_* is the tip mass; and *L* is the total length of the beam.

The boundary and continuous conditions of the structure are listed below:

(4) $$w_{1}(0,t)=0$$

(5) $$w_{1}^{\prime }(0,t)=0$$

(6) $$w_{1}(L_{1},t)=w_{2}(0,t)$$

(7) $$w_{1}^{\prime }(L_{1},t)=w_{2}^{\prime }(0,t)$$

(8) $$EI_{1}w_{1}^{\prime \prime }(L_{1},t)=EI_{2}w_{2}^{\prime \prime }(0,t)$$

(9) $$EI_{1}w_{1}^{\prime \prime \prime }(L_{1},t)=EI_{2}w_{2}^{\prime \prime \prime }\;(0,t)$$

(10) $$EI_{2}w_{2}^{\prime \prime }(L_{2},t)=0$$

(11) $$EI_{2}w_{2}^{\prime \prime \prime }\;(L_{2},t)-P\cdot w_{2}^{\prime }(L_{2},t)+M_{t}\omega_{d}^{2}w_{2}(L_{2},t)-M_{t}{\ddot{w}}_{2}(L_{2},t)=0$$

The transverse motion of the beam can be rewritten as:

(12) $$w_{k}(x_{k},t)=\sum_{i=1}^{\infty }\phi_{\;ki}(x_{k})\eta_{i}(t)$$

where *ϕ_ki_*(*x*) is the mode shape function of the *k*-th segment and the *i*-th mode; *η_i_*(*t*) is the temporal function. In this study, only the first mode was considered. The mode shape function can be further represented as:

(13) $$\phi_{\;1}(x)=C_{1}\sin(\alpha_{1}x)+C_{2}\cos(\alpha_{1}x)+C_{3}\sinh(\alpha_{2}x)+C_{4}\cosh(\alpha_{2}x)$$

(14) $$\phi_{\;2}(x)=C_{5}\sin(\alpha_{3}x)+C_{6}\cos(\alpha_{3}x)+C_{7}\sinh(\alpha_{4}x)+C_{8}\cosh(\alpha_{4}x)$$

where

(15) $$\alpha_{1}^{2}=\frac{-K_{1}^{2}+\sqrt{K_{1}^{4}+4\beta_{1}^{4}}}{2}$$

(16) $$\alpha_{2}^{2}=\frac{K_{1}^{2}+\sqrt{K_{1}^{4}+4\beta_{1}^{4}}}{2}$$

(17) $$\alpha_{3}^{2}=\frac{-K_{2}^{2}+\sqrt{K_{2}^{4}+4\beta_{2}^{4}}}{2}$$

(18) $$\alpha_{4}^{2}=\frac{K_{2}^{2}+\sqrt{K_{2}^{4}+4\beta_{2}^{4}}}{2}$$

and

(19) $$K_{k}^{2}=\frac{M_{t}\omega_{d}^{2}(r_{0}\cos\theta +L)}{EI_{k}},\;k=1,2$$

(20) $$\beta_{k}^{4}=\frac{m_{k}(\omega_{n}^{2}+\omega_{d}^{2})}{EI_{k}},\;k=1,2$$

where *ω_n_* is the resonant frequency of the beam.

The orthogonality condition was used to mass-normalize the mode shape function:

(21) $$\int_{0}^{L_{1}}\phi_{\;1m}m_{1}\phi_{\;1n}dx_{1}+\int_{0}^{L_{2}}\phi_{\;2m}m_{2}\phi_{2n}dx_{2}+\phi_{\;2m}(L_{2})M_{t}\phi_{\;2n}(L_{2})=\delta_{mn}$$

Therefore, the equations of motion can be expressed as:

(22) $$\frac{d^{2}\eta (t)}{dt^{2}}+2\zeta_{0}\omega_{n}\frac{d\eta (t)}{dt}+\omega_{n}^{2}\eta (t)+\Theta V(t)=f(t)$$

where *ζ*_0_ is the damping ratio of the system and *f*(*t*) is the normalized coefficient for excitation force caused by the gravitational force of the tip mass:

(23) $$f(t)=-\phi_{\;2}(L_{2})\cdot M_{t}g\cos\omega_{d}t$$

The piezoelectric layer can be considered as a capacitor in parallel with a current source so the electromechanical coupling equation of the circuit can be expressed as:

(24) $$-\Theta \dot{\eta }(t)+C_{p}{\dot{V}}_{p}(t)+\frac{1}{R}V_{p}(t)=0$$

where *V_p_* is the voltage across the resistive load *R*; *C_p_* is the capacitance of the piezoelectric layer. The coupling coefficient can be written as:

(25) $$\Theta =\int_{0}^{L_{1}}e_{31}b_{p}h_{pc}\frac{\partial^{3}w_{1}}{\partial x_{1}^{2}\partial t}dx_{1}$$

### 2.2. Experimental Setup

**Figure 4:**
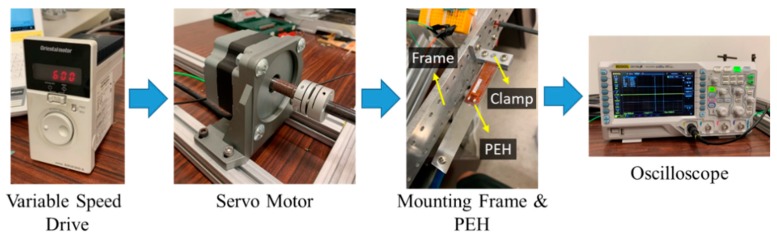
Experimental setup.

The experimental setup for the rotational tests of the PEH is depicted in Figure 4. A variable-speed drive (Oriental Motor BMUD200-A) was used to control the speed of the motor (Oriental Motor BLM5200-A), which drove a mounting frame. The PEH was fixed on the mounting frame by a clamp. The clamp was screwed on the frame, which allowed the clamp to be installed at different positions and in different orientations. The PEH was composed of a cantilever beam covered with an MFC (Macro-Fiber Composite) patch (M-2807-P2) and a tip mass was attached to the end of the beam. The MFC patch was more flexible than piezoelectric ceramics, so it was more suitable for the PEH with large deflection. The distance between the fixed end of the PEH and the rotating axis could be adjusted. The titled angle of the PEH could also be tuned to see its influence on the performance of the PEH. An oscilloscope (Rigol DS1104Z) was used to measure the voltage output of the PEH.

## 3. Results and Discussion

**Figure 5:**
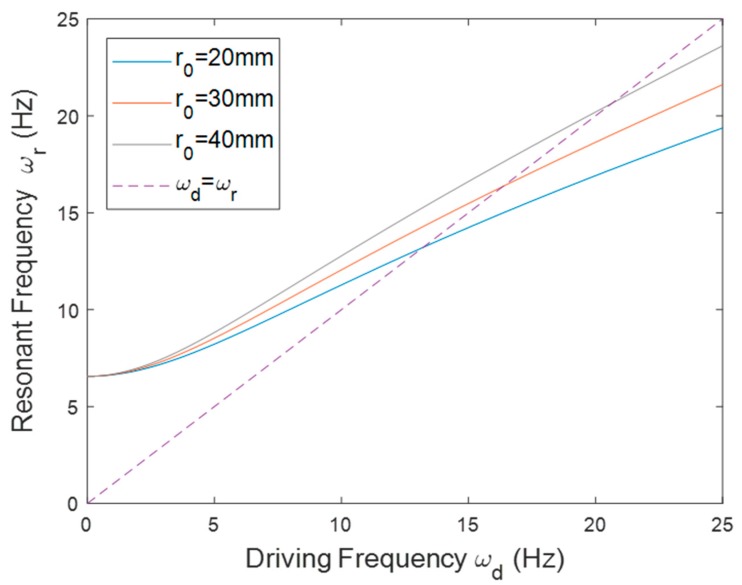
The resonant frequency of the PEH in the outward orientation with different *r*_0_.

**Figure 6:**
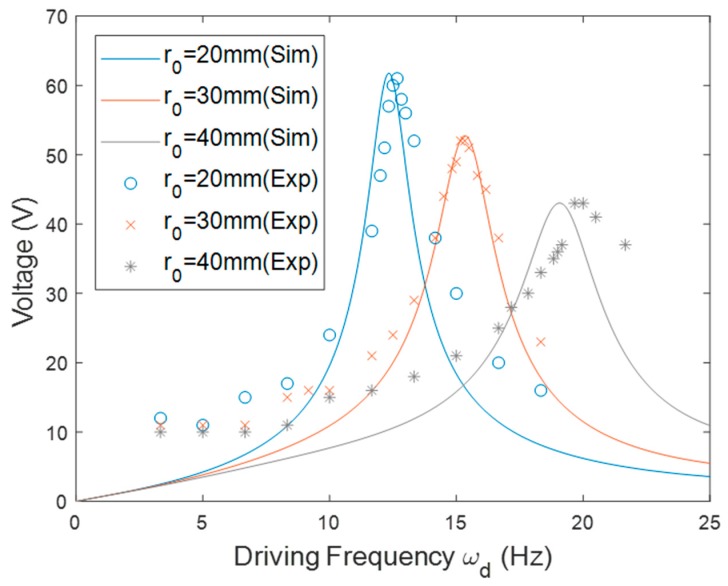
The simulation and experimental voltage responses of the PEH in the outward orientation with different *r*_0_.

The rotational PEH was first installed in the outward configuration (*θ* = 0°). The corresponding parameters are listed in Table 1. Figure 5 shows the resonant frequency of the PEH installed with different *r*_0_. In the outward orientation, the centrifugal force resulted in tensile force on the beam so the resonant frequency increased as the driving frequency rose. The matching frequencies, which are located at intersections between the solid lines and the dashed line, indicate that the resonant frequency of the PEH matched the driving frequency. Note that the resonant frequency increased as *r*_0_ was enlarged under the same driving frequency. That is because the centrifugal force caused by the tip mass was enhanced when *r*_0_ increased. The slope of the curve became steeper when *r*_0_ increased. Figure 6 depicts the simulation and experimental results of the voltage responses. The experimental results match the simulation well. The numerical peak voltage and resonant frequency were close to the experimental results. As predicted by the model, the experimental result showed that the matching frequency rose when *r*_0_ was enlarged.

**Figure 7:**
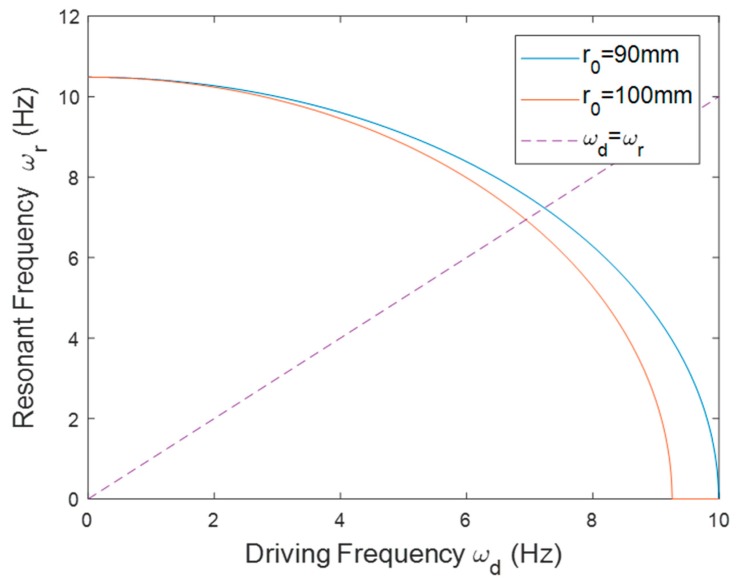
The resonant frequency of the PEH in the inward orientation with different *r*_0_.

**Figure 8:**
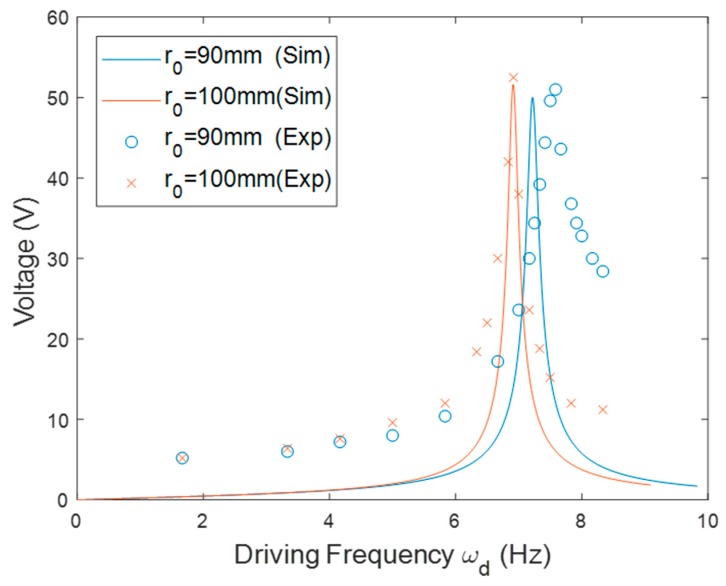
The simulation and experimental voltage responses of the PEH in the inward orientation with different *r*_0_.

For the inward configuration, the beam was slightly shortened from 75 to 72 mm to prevent the excessive amplitude of the tip displacement, since a longer beam would otherwise lead to a larger tip displacement. Figure 7 shows the resonant frequency of the PEH with different *r*_0_. The resonant frequency decreased as the driving frequency increased because the centrifugal force worked as compressive force on the beam to lower its stiffness when the beam was placed in the inward orientation. The slope of the curve became steeper when *r*_0_ increased. The matching frequency decreased when *r*_0_ was enlarged. Figure 8 depicts the frequency responses of the PEH with different *r*_0_. The resonant frequency was raised as *r*_0_ decreased in both the simulation and experimental results.

**Figure 9:**
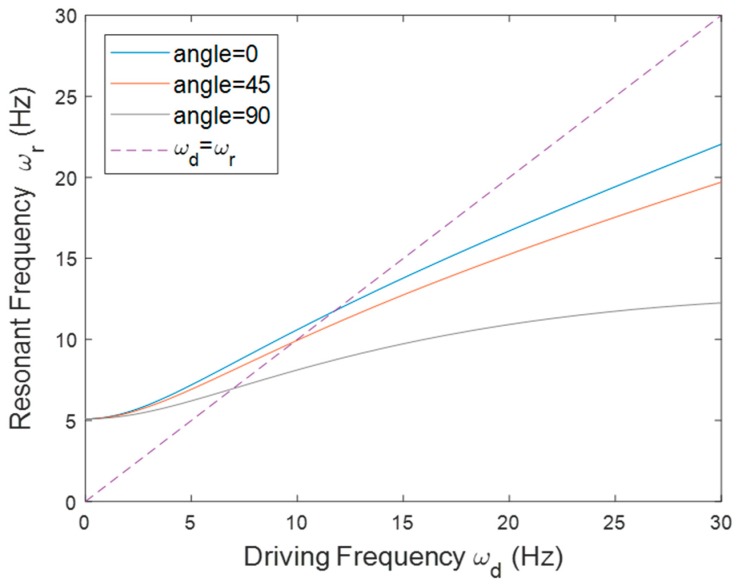
The resonant frequency of the PEH in the tilted configuration with different *θ*.

**Figure 10:**
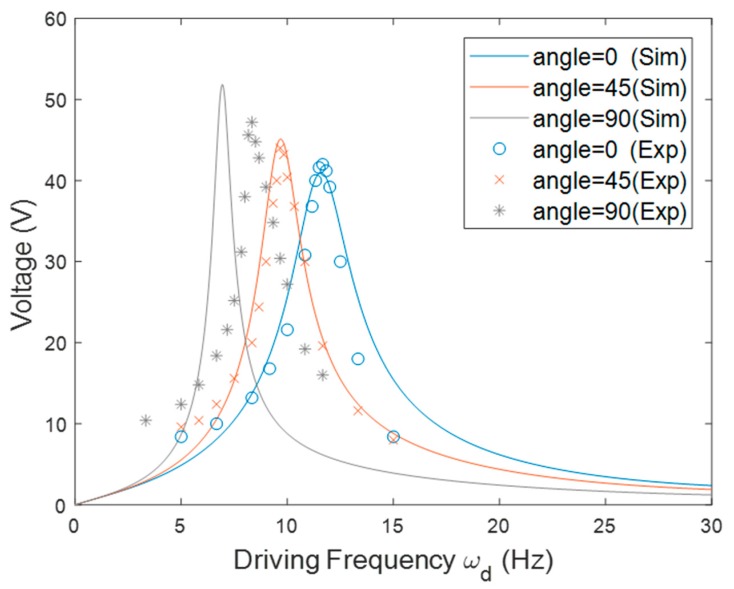
The simulation and experimental voltage responses of the PEH in the tilted orientation with different *θ*.

**Figure 11:**
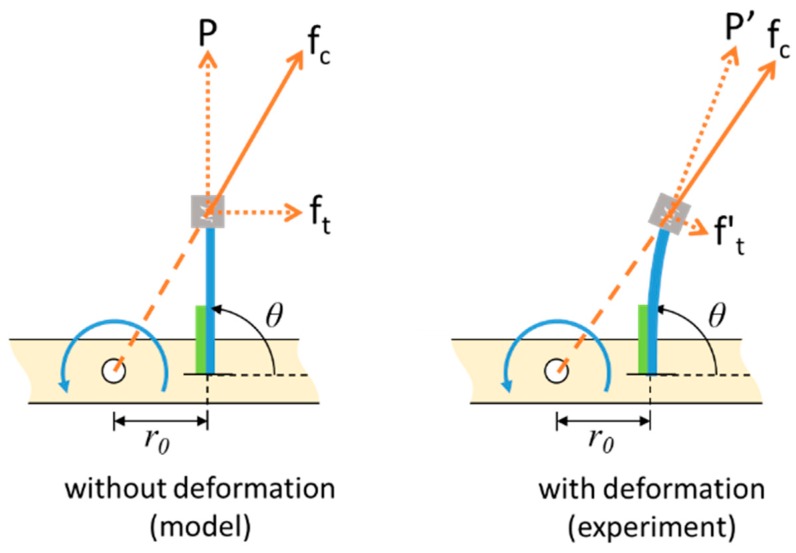
The illustration of axial load on the PEH in the tilted configuration.

Finally, the resonant frequency of the PEH in the tilted configuration is illustrated in Figure 9. The length of the beam used in this configuration was 78 mm and *r*_0_ was set to 30 mm. The PEH was tested with different tilt angles. It was expected that the tilt angle would change the magnitude of the component of centrifugal force in the longitudinal direction of the beam. The result showed that the resonant frequency decreased as the tilt angle increased, because a large tilt angle reduced the tensile axial load caused by the centrifugal force. The slope of the curve became less steep when the angle increased. A similar trend of the resonant frequency was seen in both the simulation and the experimental results shown in Figure 10. The resonant frequency reduced as the tilt angle increased in the simulation and experimental results. The peak voltage was enhanced when the resonant frequency was decreased. Note that there was a mismatch between the numerical and experimental resonant frequency when the tilt angle was 90°. The reason for this is that the component of the centrifugal force in the transverse direction of the beam made the beam deflect. The deflection would strengthen the axial load, as indicated in Figure 11, since the deflection resulted in a larger component of the centrifugal force in the axial direction. Therefore, the experimental stiffness would be higher than the stiffness estimated by the model and consequently the experimental resonant frequency would be higher than the numerical one.

## 4. Conclusions

This study examined a PEH in different orientations under rotational excitations. The installation orientation of the PEH influenced the axial component of the centrifugal force acting on the beam so the dynamics of the PEH were affected correspondingly. The dynamics of the rotational PEH was theoretically modeled and an experimental study was conducted for validation. Three configurations—outward, inward, and tilted orientations—were studied and compared. In the outward orientation, the resonant frequency of the PEH increased when the installation radius was extended, because of the increase of the centrifugal force. However, the experiment and simulation showed that the resonant frequency of the PEH in the inward orientation was raised as the installation radius was shortened. Finally, the tilt angle of the PEH had a strong influence on the resonant frequency of the PEH. When the tilt angle increased from 0° to 90°, the increase of the resonant frequency with respect to the driving frequency became less significant. In addition, as the tilt angle increased, the tensile axial load caused by the centrifugal force was reduced so the resonant frequency was lowered. Overall, this study shows that both the installation radius and the tilt angle could be used to tune the matching frequency to fit the environment for high power output. Although the installation radius was effective for tuning the matching frequency, it is constrained by the size of the rotating hub and sometimes cannot be freely adjusted. The tilt angle, however, can be used to adjust the matching frequency when the installation radius is limited.

## Figures and Tables

**Table 1:** Parameters of the PEH in the outward configuration.

Symbol	Description	Value
*L*	Length (beam)	75 mm
*b_s_*	Width (beam)	12.7 mm
*h_s_*	Thickness (beam)	0.1 mm
*ρ_s_*	Density (beam)	7930 kg/m^3^
*E_s_*	Young’s modulus (beam)	193 GPa
*L*_1_	Length (MFC)	28 mm
*b_p_*	Width (MFC)	7 mm
*h_p_*	Thickness (MFC)	0.3 mm
*ρ_p_*	Density (MFC)	5440 kg/m^3^
*E_p_*	Young’s modulus (MFC)	30.336 GPa
*M_t_*	Tip mass	3.04 g
*R*	Load resistance	1 MΩ
